# Postoperative pulmonary complications following major elective abdominal surgery: a cohort study

**DOI:** 10.1186/s13741-016-0037-0

**Published:** 2016-05-23

**Authors:** Kamlesh Patel, Fatemeh Hadian, Aysha Ali, Graham Broadley, Kate Evans, Claire Horder, Marianne Johnstone, Fiona Langlands, Jake Matthews, Prithish Narayan, Priya Rallon, Charlotte Roberts, Sonali Shah, Ravinder Vohra

**Affiliations:** West Midlands Research Collaborative, Academic Department of Surgery, School of Cancer Sciences, University of Birmingham, Birmingham, B15 2TH UK; Nottingham Oesophago-Gastric Unit, Nottingham University Hospitals, Nottingham, UK

**Keywords:** Pulmonary complications, Morbidity, Surgery, Postoperative

## Abstract

**Background:**

Postoperative pulmonary complications (PPC) are an under-reported but major cause of perioperative morbidity and mortality. The aim of this prospective, contemporary, multicentre cohort study of unselected patients undergoing major elective abdominal surgery was to determine the incidence and effects of PPC.

**Methods:**

Data on all major elective abdominal operations performed over a 2-week period in December 2014 were collected in six hospitals. The primary outcome measure of PPC at 7 days was used. Univariate and multivariate analyses were performed to investigate how different factors were associated with PPC and the effects of such complications.

**Results:**

Two hundred sixty-eight major elective abdominal operations were performed, and the internal validation showed that the data set was 99 % accurate. Thirty-two (11.9 %) PPC were reported at 7 days. PPC was more common in patients with a history of chronic obstructive pulmonary disease compared to those with no history (26.7 vs. 10.2 %, *p* < 0.001). PPC was not associated with other patient factors (e.g. age, gender, body mass index or other comorbidities), type/method of operation or postoperative analgesia. The risk of PPC appeared to increase with every additional minute of operating time independent of other factors (odds ratio 1.01 (95 % confidence intervals 1.00–1.02), *p* = 0.007). PPC significantly increase the length of hospital stay (10 vs. 3 days). Attendance to the emergency department within 30 days (27.3 vs. 10.6 %), 30-day readmission (21.7 vs. 9.9 %) and 30-day mortality (12.5 vs. 0.0 %) was higher in those with PPC.

**Conclusions:**

PPC are common and have profound effects on outcomes. Strategies need to be considered to reduce PPC.

**Electronic supplementary material:**

The online version of this article (doi:10.1186/s13741-016-0037-0) contains supplementary material, which is available to authorized users.

## Background

An estimated 234 million patients undergo major surgery worldwide every year (Weiser et al. 2008). Approximately 16 % will suffer a complication within 30 days (Kazaure et al. 2012). These include well-defined complications e.g. thromboembolic complications (NICE 2010) and surgical site infections (NICE 2013) and others which are likely to be under-reported as they do not form part of the current hospital quality measures.

One set of under-reported complications are postoperative pulmonary complications (PPC). These include a spectre of clinical conditions. PPC includes postoperative hypoxia, atelectasis, bronchospasm, pulmonary infection, pulmonary infiltrate, aspiration pneumonitis, acute respiratory distress syndrome, pleural effusions and pulmonary oedema (Arozullah et al. 2000). Depending on the severity, these can be self-limiting, require ward-based interventions e.g. antibiotics or physiotherapy, or readmission to critical care, reintubation and even death.

Some estimates suggest that the incidence of PPC is anywhere between 5 and 40 % of patients following surgeries involving the abdomen (Seiler et al. 2009; Hemmes et al. 2014; Niggebrugge et al. 1999; Treschan et al. 2012). PPC is associated with a 30-day mortality of 18 % compared with 2.5 % for those without PPC (Khuri et al. 2005). Even after risk adjustment, at 5 years post surgery, PPC is associated with a 66 % lower survival (Khuri et al. 2005). In those who do survive, the limited available evidence suggests a detrimental effect of PPC on early and late health-related quality of life (Thompson et al. 2006). Following major elective abdominal surgery, PPC results in six to nine extra hospital days and costs the healthcare system an additional $30,000 per patient (Dimick et al. 2004).

This data relates to studies conducted over 10 years ago. The incidence and effects of PPC may have changed with the advances in perioperative anaesthetic techniques e.g. non-invasive positive pressure ventilation, pain control adjuncts and enhanced recovery protocols (Hemmes et al. 2014; Kehlet and Wilmore 2008). The aim of this prospective multicentre cohort study of unselected patients undergoing major elective abdominal surgery was to determine the incidence and effects of PPC.

## Methods

Over the past 8 years, trainee-led networks in the UK have adopted a collaborative approach to deliver prospective population-level data collections and measure patient, disease, surgical and hospital variables with short-term endpoints such as readmissions and complications (Bhangu et al. 2013). Using these networks, a prospective, multicentre cohort study across six hospitals in the UK was conducted over a 2-week period in December 2014.

### Inclusion and exclusion criteria

All patients over the age of 18 years undergoing major (defined as a postoperative hospital stay of > 1 day) elective surgery (patients admitted either the day of surgery or the night before) in the study period were included. Consecutive patients undergoing benign and cancer resections on the stomach, liver, pancreas, biliary tree, small bowel, colon, rectum, bladder, kidneys and abdominal aorta were included here. Organ transplantation and emergency operations were excluded. Cholecystectomy was also excluded as the majority are performed as a day case procedure.

### Primary outcome

The primary outcome measure of PPC at 7 days was used (Additional file 1: Table S1). Demographic, intraoperative and postoperative data at day 30 were collected (Additional file 1: Table S2). The definitions for all data were derived from two recent randomised controlled trials (The PROVE Network Investigators 2014; Futier et al. 2013). Patients were investigated and diagnosed with respiratory complications as per ‘routine care’ at each institution.

### Explanatory variables

Demographic and intraoperative data were collected here as potential explanatory variables for PPC at day 7 (Additional file 1: Table S2). Further, postoperative data at day 30 were also collected (Additional file 1: Table S2).

### Data validation

To standardise data quality, a quality assurance programme has been developed for previous studies (Vohra et al. 2015). This included a detailed study protocol, a pilot phase, and a requirement for a minimum of 95 % data completeness at submission. Case ascertainment and data accuracy were further validated by independent investigators at selected hospitals, who checked data correctness from 10 % of patients against original medical records. These independent investigators were not involved in the original data collection.

### Ethical approval

The protocol did not require research registration or consent from patients as only anonymous observational data were collected. Data collection was entirely independent of patient management, and therefore, patient management was not altered as a result of the study. This was confirmed by the online National Research Ethics Service (NRES) decision tool (http://www.hra-decisiontools.org.uk/research/) used to determine whether a study requires review from a research ethics committee in the UK National Health Service (NHS). This decision was further supported by the Research and Development Director at University Hospitals Birmingham NHS Foundation Trust, UK. The study was registered as a ‘clinical audit’ or ‘service evaluation’ at each participating hospital under the supervision of a named senior investigator (consultant or attending surgeon).

### Statistical analysis

Data were collected and analysed in clinically relevant categories. Univariate and multivariate analyses, including factors with a *p* < 0.05 on univariate analysis, were performed to investigate how different factors were associated with PPC and the sequelae of such complications. Missing data for predictor values were replaced using the multiple imputation method to create five imputed datasets; all predictor and outcome variables will be entered into the predictive models for imputation. Statistical analyses were conducted using SPSS v21 (IBM, Armonk, NY, USA). Statistical significance was set at *p* < 0.050. The report of this study was prepared in accordance to the guidelines set by the Strengthening the Reporting of Observational Studies in Epidemiology (STROBE) statement for observational studies (Von Elm et al. 2007).

## Results

### Demographics

Over the 2-week period, a total of 268 consecutive major elective abdominal operations were performed in the six hospitals. Case ascertainment and accuracy of collected data were above 99 and 98 %, respectively, when compared with a 20 % sample checked independently against the original medical records. Missing data were 0.8 % within the entire dataset. General demographic data is shown in Table 1. Median age of the cohort was 66 years, 61.9 % were male, 53.7 % were ASA grade 1 or 2 and 31 % were having a cancer operation. In addition, it is notable that 84.3 % were administered perioperative antibiotics, 63.1 % had open operations and despite all operations being classed as major operations, elective critical care use was 19.4 %. Endotracheal tubes were mainly used reflecting the duration and grade of the operations performed.

**Table 1:** General demographics

*n*	268
Age (years, IQR)	66 (53–75)
Male	166 (61.9)
Body mass index (kilograms/metres^2^, IQR)	27.3 (24.0–31.2)
American Society of Anesthesiologists physical status classification system	
1	32 (11.9)
2	112 (41.8)
3	71 (26.5)
4	7 (2.6)
Unknown	46 (17.2)
Current smoker	42 (15.7)
Chronic obstructive pulmonary disease	30 (11.2)
Previous cerebrovascular accident	23 (8.6)
Urea (milligrams per decilitre, IQR)	5.4 (4.5–7.0)
Proton pump inhibitor	89 (33.2)
Steroids	16 (6.0)
Cancer operation	83 (31.0)
Type of operation	
Gastric	28 (10.4)
Hepatobiliary/pancreatic	19 (7.1)
Small bowel	13 (4.9)
Colorectal	76 (28.4)
Urological	57 (21.3)
Vascular	62 (23.1)
Other	13 (4.9)
Perioperative antibiotics	226 (84.3)
Type of intubation	
Laryngeal mask airway	60 (22.4)
Cuffed/uncuffed endotracheal tube	207 (77.2)
Unknown	1 (0.4)
Method of operation	
Laparoscopic	77 (28.7)
Laparoscopic-assisted	6 (2.2)
Laparoscopic converted to open	8 (3.0)
Open	169 (63.1)
Endovascular	8 (3.0)
Bowel resection	74 (27.6)
Nasogastric tube	9 (3.4)
Duration of surgery (minutes, IQR)	145 (87–210)
Elective critical care admission	52 (19.4)
Analgesia use in the first 24 h	
Epidural	40 (14.9)
Patient controlled analgesia	50 (18.7)
Wound catheter	2 (0.7)
Oral analgesia only	173 (64.6)
Unknown	3 (1.1)
Change in analgesia strategy in the first 24 h	171 (63.8)
Incentive spirometer	3 (1.1)

### Outcomes at day 7 and 30 (Table 2)

**Table 2:** Selected outcomes within seven and thirty days

At 7 days	
Severe hypoxia	6 (2.2)
Bronchospasm	1 (0.4)
Suspected pulmonary infection	24 (9.0)
Pulmonary infiltrate	11 (4.1)
Aspiration pneumonitis	2 (0.7)
Acute respiratory distress syndrome	2 (0.7)
Atelectasis	13 (4.9)
Pleural effusion	10 (3.7)
Pulmonary oedema	6 (2.2)
Any postoperative pulmonary complication	32 (11.9)
Readmission to critical care	7 (2.6)
Reintubation	5 (1.9)
Length of hospital stay (days, IQR)	4 (1–7)
At 30 days	
Accident and emergency attendance	22 (8.2)
Readmission	46 (17.2)
All complications	59 (22.0)
Non-pulmonary complications	37 (13.8)
Mortality	4 (1.5)

A total of 32 (11.9 %) PPC were reported at 7 days, and suspected pulmonary infection was the most common (*n* = 24, 9 %). The median length of hospital stay for the cohort was 4 days, and 30-day mortality was 1.5 % (*n* = 4). Readmissions at 30 days were 17 % which reflects the grade of operations performed.

### Effect of pre and perioperative factors (Tables 3 and 4)

**Table 3:** Effect of perioperative variables on PPC

	PPC No. (%)	Yes (%)	
*n*	236	32	
Age (years, IQR)	67 (53–76)	60 (57–67)	0.404
Gender			0.647
Male	145 (61.4)	21 (65.6)	
Female	91 (38.6)	11 (34.4)	
Body mass index (kilograms/metres^2^, IQR)	27.3 (23.8–30.5)	27.1 (25.6–29.9)	0.742
American Society of Anesthesiologists physical status classification system			0.244
1	50 (21.2)	3 (9.4)	
2	119 (50.4)	13 (40.6)	
3	58 (24.7)	12 (37.6)	
4	6 (2.5)	2 (6.2)	
Unknown	3 (1.2)	2 (6.2)	
Current smoker	36 (15.3)	6 (18.8)	0.662
Chronic obstructive pulmonary disease	22 (9.3)	8 (25.0)	0.009
Previous cerebrovascular accident	21 (8.9)	2 (6.3)	0.603
Urea (milligrams per decilitre, IQR)	5.6 (4.5–7.3)	5.4 (4.0–9.4)	0.886
Proton pump inhibitor	80 (33.9)	9 (28.1)	0.515
Steroids	13 (5.5)	3 (9.4)	0.390
Cancer operation	67 (28.4)	15 (46.9)	0.035
Type of operation			0.270
Gastric	24 (10.2)	4 (12.5)	
Hepatobiliary/pancreatic	17 (7.2)	2 (6.3)	
Small bowel	12 (5.1)	1 (3.1)	
Colorectal	66 (28.0)	10 (31.3)	
Urological	55 (23.3)	2 (6.3)	
Vascular	52 (22.0)	10 (31.3)	
Other	10 (4.2)	3 (9.2)	
Perioperative antibiotics	200 (84.7)	26 (81.3)	0.860
Type of intubation			0.946
Laryngeal mask airway	54 (22.9)	6 (18.8)	
Cuffed/uncuffed endotracheal tube	181 (76.7)	26 (81.2)	
Unknown	1 (0.4)	0 (0.0)	
Method of operation			0.722
Laparoscopic	72 (30.5)	5 (15.6)	
Laparoscopic-assisted	5 (2.1)	1 (3.1)	
Laparoscopic converted to open	6 (2.5)	2 (6.3)	
Open	145 (61.4)	24 (75.0)	
Endovascular	8 (3.5)	0 (0.0)	
Bowel resection	61 (25.8)	13 (17.6)	0.079
Nasogastric tube	6 (2.5)	3 (9.4)	0.011
Duration of surgery (minutes, IQR)	100 (55–170)	212 (182–294)	<0.001
Elective critical care admission	33 (14.0)	19 (59.4)	<0.001
Analgesia use in the first 24 h			<0.001
Epidural	26 (11.0)	14 (43.8)	
Patient controlled analgesia	40 (16.9)	10 (31.3)	
Wound catheter	2 (0.1)	0 (0.0)	
Oral analgesia only	166 (70.3)	7 (21.8)	
Unknown	2 (0.1)	1 (3.1)	
Change in analgesia strategy in the first 24 h	148 (62.7)	23 (71.9)	0.014
Incentive spirometer	1 (0.4)	2 (6.3)	<0.001

**Table 4:** Selected factors and adjusted odds ratios for postoperative pulmonary complications

	OR (95 % CI)	*p* value
Chronic obstructive pulmonary disease	16.77 (2.56–109.88)	0.003
Cancer operation	5.13 (0.41–62.5)	0.205
Nasogastric tube	2.15 (0.33–4.01)	0.411
Duration of surgery	1.01 (1.00–1.02)	0.007
Elective critical care admission	4.45 (0.45–43.75)	0.200
Analgesia use in the first 24 h		
Epidural	Reference	
Patient controlled analgesia	0.83 (0.05–15.26)	0.898
Wound catheter	3.05 (0.40–22.98)	0.279
Oral analgesia only	2.56 (0.23–10.54)	0.636
Change in analgesia strategy in the first 24 h	0.24 (0.02–2.70)	0.247
Incentive spirometer	2.93 (0.12–23.83)	0.782

Risk factors for PPC by day 7 were a history of chronic obstructive pulmonary disease, undergoing an operation for a malignancy and a postoperative nasogastric tube. In addition, the duration of surgery associated with PPC at day 7 as was the intraoperative analgesia strategy and a change to this strategy in the first 24 h. PPC was not associated with age, gender, body mass index, other comorbidities, smoking or type or method of operation (open vs. laparoscopic).

When these significant factors were included in a multivariate model, chronic obstructive pulmonary disease was independently associated with development of a PPC (Table 4). Further, the risk of PPC appeared to increase with every additional minute of operating time independent of other factors (odds ratio 1.01 (95 % confidence intervals 1.00–1.02), *p* = 0.007).

### Impact of PPC at day 30

The four postoperative deaths occurred exclusively in patients who developed PPC. PPC increased the median length of hospital stay by 7 days, attendance to the emergency department by 38.8 % and readmissions by 45.6 % compared to if no PPC occurred (Table 5).

**Table 5:** Outcomes and postoperative pulmonary complications (PPC)

	PPC		
	No (%)	Yes (%)	
Length of hospital stay (days, IQR)	3 (1–6)	10 (7–16)	< 0.001
At 30 days			
Accident and Emergency attendance	26 (11.0)	6 (27.3)	0.014
Readmission	22 (9.3)	10 (21.7)	0.014
Mortality	0 (0.0)	4 (12.5)	< 0.001

## Discussion

This prospective multicentre cohort study investigated pulmonary complications in an unselected cohort following major elective abdominal surgery. The data set was internally and independently validated. The incidence of PPC was 11.9 %. PPC were associated with preoperative and intraoperative risk factors. Despite the small cohort, the development of PPC had a significant impact on length of hospital stay and 30-day outcomes. The mortalities within our cohort were exclusively in patients with a PPC. Cause of death was not examined as part of the study, and thus, we were unable to infer causality; however, length of stay and outcomes data support the morbidity secondary to PPC.

PPC have been estimated by both retrospective cohort studies and in randomised controlled trials (Seiler et al. 2009; Hemmes et al. 2014; Niggebrugge et al. 1999; Treschan et al. 2012; Holte et al. 2007; Squadrone et al. 2005; Pöpping et al. 2008). This has produced a wide disparity in the reported incidence of PPC. The definitions of PPC used in this cohort study were derived from two recent randomised controlled trials investigating differences in tidal volume settings in patients undergoing major elective abdominal surgery performed by an open procedure (Hemmes et al. 2014; Futier et al. 2013). These two studies estimated the incidence of PPC between 20 and 40 %. The incidence estimated in the data presented here was lower than previously described despite high internal study validity. This may be explained by differences in patients’ risk factors (e.g. smoking status) and surgical procedures between the unselected cohort studied here and those randomised in previous studies (Hemmes et al. 2014; Futier et al. 2013). Another explanation may be the high numbers of laparoscopic and laparoscopic-assisted procedures performed in this series. However, these approaches are linked with longer operating times, which in this study and previous studies are associated with a higher incidence of PPC (Canet et al. 2010).

PPC was associated with worse 30-day outcomes in this study. The most striking impact of PPC was the effect on hospital length of stay. Median hospital length of stay was extended from 3 to 10 days if a PPC occurred. This is similar to data from 2001 to 2002 from the National Surgical Quality Improvement Project (NSQIP) in the USA (Dimick et al. 2004) which demonstrated that a PPC resulted in an additional six hospital days. Other infectious, cardiovascular and thromboembolic complications resulted in an additional three, two and three hospital days, respectively. In addition, PPC were associated with three times higher healthcare costs compared to other complications.

Other studies have shown continued demonstrable effects of a PPC 5 years following the index event (Khuri et al. 2005). PPCs have a multifactorial aetiology including ventilation-perfusion mismatch and hypoxemia which is a consequence of general anaesthesia, postoperative pain, diaphragmatic dysfunction, decreased chest wall compliance and depressed airway reflexes (Canet et al. 2010; Gazarian 2006; Ebell 2007). This is confounded by bacterial entry into the lower respiratory tract by aspiration of oral and pharyngeal pathogens at the time of intubation and continued leakage of secretions containing bacteria around the endotracheal tube cuff when patients are ventilated for prolonged periods (days to weeks) (du Moulin et al. 1982; Cook et al. 1998; American Thoracic Society, Infectious Diseases Society of America 2005). Colonisation of the lower respiratory tract can overwhelm the patients’ mechanical, humoral, and cellular defences to establish infection following surgery (Craven and Steger 1996). Chronic airway inflammation, copious airway secretions and use of preoperative steroids causing immunosuppression may be to blame for increased PPC in patients with COPD (Banerjee et al. 2004).

A patient safety summit statement recently recommended that PPC should be a measure of healthcare quality as it is likely to require a multifaceted and multidisciplinary approach to reduce the incidence (Shander et al. 2011). The definitions used here were monitored successfully by junior surgeons with high internal study validity using prospective cross-sectional methodology described previously (Vohra et al. 2015). These data fields could be used to provide ongoing monitoring of PPC incidence. High incidences of PPC, following patient stratification and risk adjustment, may be used to indicate deficiencies in the perioperative care of patients undergoing major surgery.

## Conclusions

This study highlights the frequency at which PPC occur and their subsequent effects on short-term outcomes. Other studies have shown further implications for long-term patient morbidity.

The development of any PPC is associated with significant morbidity reflected in worse 7- and 30-day outcomes as demonstrated here. Standardised care bundles and other novel strategies need to be considered to reduce PPC across all surgical patients.

## Additional file

Additional file 1: Supplementary tables. (DOCX 23 kb)

## Abbreviations

COPD: chronic obstructive pulmonary disease
PPC: postoperative pulmonary complications

## References

[CR1] American Thoracic Society, Infectious Diseases Society of America (2005). Guidelines for the management of adults with hospital-acquired, ventilator-associated, and healthcare-associated pneumonia. Am J Respir Crit Care Med.

[CR2] Arozullah AM, Daley J, Henderson WG, Khuri SF (2000). Multifactorial risk index for predicting postoperative respiratory failure in men after major noncardiac surgery. The National Veterans Administration Surgical Quality Improvement Program. Ann Surg.

[CR3] Banerjee D, Khair OA, Honeybourne D (2004). Impact of sputum bacteria on airway inflammation and health status in clinical stable COPD. Eur Respir J.

[CR4] Bhangu A, Kolias AG, Pinkney T, Hall NJ, Fitzgerald JE (2013). Surgical research collaboratives in the UK. Lancet.

[CR5] Canet J, Gallart L, Gomar C, Paluzie G, Vallès J, Castillo J (2010). Prediction of postoperative pulmonary complications in a population-based surgical cohort. Anesthesiology.

[CR6] Cook D, De Jonghe B, Brochard L, Brun-Buisson C (1998). Influence of airway management on ventilator-associated pneumonia: evidence from randomized trials. JAMA.

[CR7] Craven DE, Steger KA (1996). Nosocomial pneumonia in mechanically ventilated adult patients: epidemiology and prevention in 1996. Semin Respir Infect.

[CR8] Dimick JB, Chen SL, Taheri PA, Henderson WG, Khuri SF, Campbell DA (2004). Hospital costs associated with surgical complications: a report from the private-sector National Surgical Quality Improvement Program. J Am Coll Surg.

[CR9] du Moulin GC, Paterson DG, Hedley-Whyte J, Lisbon A (1982). Aspiration of gastric bacteria in antacid-treated patients: a frequent cause of postoperative colonisation of the airway. Lancet.

[CR10] Ebell MH (2007). Predicting postoperative pulmonary complications. Am Fam Physician.

[CR11] Futier E, Constantin JM, Paugam-Burtz C, Pascal J, Eurin M (2013). A trial of intraoperative low-tidal-volume ventilation in abdominal surgery. N Engl J Med.

[CR12] Gazarian PK (2006). Identifying risk factors for postoperative pulmonary complications. AORN J.

[CR13] Hemmes SN, Gama de Abreu M, Pelosi P, Schultz MJ, PROVE Network Investigators for the Clinical Trial Network of the European Society of Anaesthesiology (2014). High versus low positive end-expiratory pressure during general anaesthesia for open abdominal surgery (PROVHILO trial): a multicentre randomised controlled trial. Lancet.

[CR14] Holte K, Foss NB, Andersen J, Valentiner L, Lund C, Bie P (2007). Liberal or restrictive fluid administration in fast-track colonic surgery: a randomized, double-blind study. Br J Anaesth.

[CR15] Kazaure HS, Roman SA, Sosa JA (2012). Association of postdischarge complications with reoperation and mortality in general surgery. Arch Surg.

[CR16] Kehlet H, Wilmore DW (2008). Evidence-based surgical care and the evolution of fast-track surgery. Ann Surg.

[CR17] Khuri SF, Henderson WG, DePalma RG, Mosca C, Healey NA, Kumbhani DJ (2005). Determinants of long-term survival after major surgery and the adverse effect of postoperative complications. Ann Surg.

[CR18] NICE. Surgical site infection. NICE quality standard 49. 2013. Available online at http://www.nice.org.uk/guidance/qs49/resources/guidance-surgical-site-infection-pdf. Accessed 12 jan 2015.

[CR19] NICE. Venous thromboembolism prevention quality standard. NICE quality standard 3. 2010. Available online at http://www.nice.org.uk/guidance/qs3/resources/guidance-venous-thromboembolism-prevention-quality-standard-pdf. Accessed 12 jan 2015.

[CR20] Niggebrugge AH, Trimbos JB, Hermans J, Steup WH, Van De Velde CJ (1999). Influence of abdominal-wound closure technique on complications after surgery: a randomised study. Lancet.

[CR21] Pöpping DM, Elia N, Marret E, Remy C, Tramèr MR (2008). Protective effects of epidural analgesia on pulmonary complications after abdominal and thoracic surgery: a meta-analysis. Arch Surg.

[CR22] Seiler CM, Deckert A, Diener MK, Knaebel HP, Weigand MA, Victor N (2009). Midline versus transverse incision in major abdominal surgery: a randomized, double-blind equivalence trial (POVATI: ISRCTN60734227). Ann Surg.

[CR23] Shander A, Fleisher LA, Barie PS, Bigatello LM, Sladen RN, Watson CB (2011). Clinical and economic burden of postoperative pulmonary complications: patient safety summit on definition, risk reducing interventions, and preventive strategies. Crit Care Med.

[CR24] Squadrone V, Coha M, Cerutti E, Schellino MM, Biolino P, Occella P (2005). Continuous positive airway pressure for treatment of postoperative hypoxemia: a randomized controlled trial. JAMA.

[CR25] The PROVE Network Investigators (2014). High versus low positive end-expiratory pressure during general anaesthesia for open abdominal surgery (PROVHILO trial): a multicentre randomised controlled trial. Lancet.

[CR26] Thompson DA, Makary MA, Dorman T, Pronovost PJ (2006). Clinical and economic outcomes of hospital acquired pneumonia in intra-abdominal surgery patients. Ann Surg.

[CR27] Treschan TA, Kaisers W, Schaefer MS, Bastin B, Schmalz U, Wania V (2012). Ventilation with low tidal volumes during upper abdominal surgery does not improve postoperative lung function. Br J Anaesth.

[CR28] Vohra RS, Spreadborough P, Johnstone M, Marriott P, Bhangu A, Alderson D (2015). West Midlands Research Collaborative. Protocol for a multicentre, prospective, population-based cohort study of variation in practice of cholecystectomy and surgical outcomes (The CholeS study). BMJ Open.

[CR29] Von Elm E, Altman DG, Egger M, Pocock SJ, Gøtzsche PC, Vandenbroucke JP (2007). Strengthening the reporting of observational studies in epidemiology (STROBE) statement: guidelines for reporting observational studies. BMJ.

[CR30] Weiser TG, Regenbogen SE, Thompson KD, Haynes AB, Lipsitz SR, Berry WR (2008). An estimation of the global volume of surgery: a modelling strategy based on available data. Lancet.

