# Supplementary material for: Postoperative pulmonary complications following major elective abdominal surgery: a cohort study
Source: Perioper Med (Lond). 2016 May 23;5:10. doi: 10.1186/s13741-016-0037-0 (PMC4877761; doi:10.1186/s13741-016-0037-0)
Supplement: Additional file 1: — Supplementary tables. (DOCX 23 kb) [file 13741_2016_37_MOESM1_ESM.docx]

**Additional file 1**

Supplementary table 1

Definitions of pulmonary postoperative complications

| Hypoxaemia | PaO2 < 60 mmHg or SpO2 < 90% in room air, but responding to supplemental oxygen (excluding hypoventilation) |
| --- | --- |
| Severe Hypoxia | Need for non–invasive or invasive mechanical ventilation or a PaO2 < 60 mmHg or SpO2 < 90% despite supplemental oxygen (excluding hypoventilation) |
| Bronchospasm | Defined as newly detected expiratory wheezing treated with bronchodilators |
| Suspected pulmonary infection | In case patient receives antibiotics and meets at least one of the following criteria: new or changed sputum, new or changed lung opacities on chest X–ray when clinically indicated, tympanic temperature > 38·3°C, WBC count > 12 x109/L |
| Pulmonary infiltrate | Chest X–ray demonstrating mono or bilateral infiltrate |
| Aspiration pneumonitis | Defined as respiratory failure after the inhalation of regurgitated gastric contents |
| Acute Respiratory Distress Syndrome | By the consensus criteria (only in case of non–invasive or invasive mechanical ventilation) |
| Atelectasis | Suggested by lung opacification with shift of the mediastinum, hilum, or hemi-diaphragm towards the affected area, and compensatory over-inflation in the adjacent non-atelectatic lung |
| Pleural effusion | Chest X–ray demonstrating blunting of the costophrenic angle, loss of the sharp silhouette of the ipsilateral hemi-diaphragm in upright position, evidence of displacement of adjacent anatomical structures, or (in supine position) a hazy opacity in one hemi–thorax with preserved vascular shadows |
| Pulmonary oedema | Defined as clinical signs of congestion, including dyspnoea, oedema, rales and jugular venous distention, with the chest X–ray demonstrating increase in vascular markings and diffuse alveolar interstitial infiltrates |

Supplementary table 2

| **Pre-operative data fields** | |
| --- | --- |
| Age | In years |
| Gender | Male, female |
| Body mass index | Individual’s body mass will be subclassified as:   - Underweight (<17.9 kg/m2) - Normal (18.0–24.9 kg/m2) - Overweight (25.0–29.9 kg/m2) - Moderate obesity (30.0–34.9 kg/m2) - Severe obesity (35.0–39.9 kg/m2) - Very severe obesity (>40.0 kg/m2) |
| ASA | Classified as: 1. A normal healthy patient 2. A patient with mild systemic disease 3. A patient with severe systemic disease 4. A patient with severe systemic disease that is a constant threat to life 5. A moribund patient who is not expected to survive without the operation |
| Current smoker | Yes/ No |
| Chronic obstructive pulmonary disease | Yes/ No |
| Previous cerebrovascular accident | Yes/ No |
| Pre-operative Urea | Milligrams per decilitre |
| Proton pump inhibitor use pre-operatively | Yes/ No |
| Steroid use pre-operatively | Yes/ No |
| **Intra-operative data fields** | |
| Type of operation | Gastric  Hepatobiliary / pancreatic  Small bowel  Colorectal  Urological  Vascular  Other |
| Perioperative antibiotics | Yes/ No |
| Type of intubation | Laryngeal mask airway  Cuffed / uncuffed endotracheal tube |
| Method of operation | Laparoscopic  Laparoscopic-assisted  Laparoscopic converted to open  Open  Endovascular |
| Bowel resection | Yes/ No |
| Intra-operative NG tube insertion | Yes/ No |
| Duration of surgery | Minutes |
| **Post-operative data fields** | |
| Elective critical care admission | Yes/ No |
| Analgesia use in the 1^st^ 24 hours | Epidural  Patient controlled analgesia  Wound Catheter  Oral analgesia only  Unknown |
| Change in analgesia strategy in the 1^st^ 24 hours | Yes/ No |
| Use of incentive spirometer post-operatively | Yes/ No |
| Readmission to critical care | Yes/ No |
| Reintubation | Yes/ No |
| Length of hospital stay | Days |
| **Post-operative data following discharge** | |
| Accident and Emergency attendance | Yes/ No |
| Readmission | Yes/ No |
| All Complications | Yes/ No |
| Non-pulmonary complications | Yes/ No |
| Mortality | Yes/ No |
